# Complete Genome Sequence of Klebsiella pneumoniae Myophage Mulock

**DOI:** 10.1128/MRA.01338-19

**Published:** 2019-11-14

**Authors:** Lorna Min, Lauren Lessor, Chandler O’Leary, Rachele Bonasera, Jason Gill, Mei Liu

**Affiliations:** a Center for Phage Technology, Texas A&M University, College Station, Texas, USA; Queens College

## Abstract

Klebsiella pneumoniae is an opportunistic pathogen associated with hospital-acquired infections. This report describes the complete genome of the K. pneumoniae myophage Mulock, which appears to be a temperate myophage distantly related to other *Klebsiella* myophages in morphogenesis genes and is partially syntenic with the canonical *Escherichia* phage lambda in genes encoding lambda-like functions.

## ANNOUNCEMENT

Klebsiella pneumoniae is an opportunistic pathogen associated with outbreaks in hospital-acquired infections such as urinary tract infections, pneumonia, and meningitis (1). The increase in antibiotic resistance has complicated treatment for this pathogen and led to an increase in associated deaths (2, 3). Studying bacteriophages infecting K. pneumoniae may help us to understand the biology of this organism and develop treatment strategies.

Phage Mulock was isolated from a wastewater sample collected from Brazos County, TX, in 2015 using a deidentified K. pneumoniae clinical isolate as the host. Host bacteria were cultured on tryptic soy broth or agar (Difco) at 37°C with aeration. Phages were cultured and propagated using the soft-agar overlay method (4). The phage was identified as a myophage using negative-stain transmission electron microscopy performed at the Texas A&M University Microscopy and Imaging Center as described previously (5). Phage genomic DNA was prepared using a modified Promega Wizard DNA cleanup kit protocol as described previously (5). Pooled indexed DNA libraries were prepared using the Illumina TruSeq Nano LT kit, and the sequence was obtained from the Illumina MiSeq platform using the MiSeq V2 500-cycle reagent kit following the manufacturer’s instructions, producing 1,069,908 paired-end 250-bp reads for the index containing the phage Mulock genome. FastQC 0.11.5 (https://www.bioinformatics.babraham.ac.uk/projects/fastqc/) was used to quality control the reads. The reads were trimmed with FastX Toolkit 0.0.14 (http://hannonlab.cshl.edu/fastx_toolkit/download.html) before being assembled using SPAdes 3.5.0 (6). Contig completion was confirmed with PCR using primers (5′-TGCAGTCAATTGGTGGAAGA-3′, 5′-ATGGCATGGCGATAGGAATA-3′) facing off the ends of the assembled contig and Sanger sequencing of the resulting product, with the contig sequence manually corrected to match the resulting Sanger sequencing read. GLIMMER 3.0 (7) and MetaGeneAnnotator 1.0 (8) were used to predict protein coding genes with manual verification, and tRNA genes were predicted with ARAGORN 2.36 (9). Rho-independent termination sites were identified via TransTermHP (http://transterm.cbcb.umd.edu/). Sequence similarity searches were done using BLASTp 2.2.28 (10) with a maximum expectation cutoff of 0.001 against the NCBI nonredundant (nr), UniProt Swiss-Prot (11), and TrEMBL databases. InterProScan 5.15-54.0 (12), LipoP (13), and TMHMM v2.0 (14) were used to predict protein function. All analyses were conducted at default settings via the CPT Galaxy (15) and WebApollo (16) interfaces (https://cpt.tamu.edu/galaxy-pub).

Phage Mulock was assembled at 30.2-fold coverage into a complete genome of 43,727 bp. Mulock appears to be a temperate myophage distantly related to other *Klebsiella* phages; at the DNA level, as determined by a BLASTn search against the NCBI nucleotide (nt) database (E value, <0.001), Mulock is related in its left end, encoding morphogenetic functions, to other *Klebsiella* phages (such as those deposited under GenBank accession no. MK433579 and MK448230) and prophages found integrated into *Klebsiella* genomes (such as that under GenBank accession no. CP034200). In its central and rightward regions, phage Mulock is partially syntenic with the canonical *Escherichia* phage lambda (GenBank accession no. NC_001416) in genes encoding lambda-like functions, including integration, generalized recombination, lysogeny control, DNA replication, and lysis. The homologs to lambda Exo, Bet, cII, O, P, and NinG were identified. Mulock, however, utilizes lysis proteins of classes other than lambda. Mulock possesses a class II holin, a signal-arrest-release (SAR) endolysin, and an overlapping spanin pair. Mulock is not closely related to other phages at the protein level based on a BLASTp search against the NCBI nr database.

### Data availability.

The genome sequence of phage Mulock was submitted to GenBank under the accession no. MN098327. The associated BioProject, SRA, and BioSample accession numbers are PRJNA222858, SRR8771457, and SAMN11234224, respectively.
